# Identification of four serum miRNAs as potential markers to screen for thirteen cancer types

**DOI:** 10.1371/journal.pone.0269554

**Published:** 2022-06-10

**Authors:** Joe W. Chen, Joseph Dhahbi

**Affiliations:** California University of Science and Medicine, Colton, CA, United States of America; University of Nebraska Medical Center, UNITED STATES

## Abstract

**Introduction:**

Cancer consistently remains one of the top causes of death in the United States every year, with many cancer deaths preventable if detected early. Circulating serum miRNAs are a promising, minimally invasive supplement or even an alternative to many current screening procedures. Many studies have shown that different serum miRNAs can discriminate healthy individuals from those with certain types of cancer. Although many of those miRNAs are often reported to be significant in one cancer type, they are also altered in other cancer types. Currently, very few studies have investigated serum miRNA biomarkers for multiple cancer types for general cancer screening purposes.

**Method:**

To identify serum miRNAs that would be useful in screening multiple types of cancers, microarray cancer datasets were curated, yielding 13 different types of cancer with a total of 3352 cancer samples and 2809 non-cancer samples. The samples were divided into training and validation sets. One hundred random forest models were built using the training set to select candidate miRNAs. The selected miRNAs were then used in the validation set to see how well they differentiate cancer from normal samples in an independent dataset. Furthermore, the interactions between these miRNAs and their target mRNAs were investigated.

**Result:**

The random forest models achieved an average of 97% accuracy in the training set with 95% bootstrap confidence interval of 0.9544 to 0.9778. The selected miRNAs were hsa-miR-663a, hsa-miR-6802-5p, hsa-miR-6784-5p, hsa-miR-3184-5p, and hsa-miR-8073. Each miRNA exhibited high area under the curve (AUC) value using receiver operating characteristic analysis. Moreover, the combination of four out of five miRNAs achieved the highest AUC value of 0.9815 with high sensitivity of 0.9773, indicating that these miRNAs have a high potential for cancer screening. miRNA-mRNA and protein-protein interaction analysis provided insights into how these miRNAs play a role in cancer.

## Introduction

Cancer has consistently been one of the most common causes of death in the United States, precisely the second leading cause in 2020 [1]. Therefore, effective cancer screening and early detection are crucial for improving healthcare outcomes [2, 3]. However, many of the current standards for cancer screening lack sufficient sensitivity and specificity, and many of the screening modalities are invasive [3]. In addition, many cancers such as ovarian cancer and pancreatic cancer are known to be deadly because of late-stage discovery [4, 5]. With the stable nature of miRNAs, circulating serum miRNAs can serve as a minimally invasive alternative or supplement the current standard for cancer screening [6, 7].

Many miRNAs have already been reported to be promising biomarkers for certain types of cancer. For instance, plasma miR-145, miR-20a, miR-21, and miR-223 have been shown to be biomarkers for screening of early-stage non-small cell lung cancer [8, 9]. Similarly, serum miR-21 and other miRNAs are also found to be differentially regulated in glioma compared to healthy controls [10]. Other different panels of miRNAs can be used in early-stage breast, colorectal, and other cancer diagnoses as well [11–18]. However, some of these reported miRNAs may be non-specific; many miRNAs while being important in one cancer are also altered in other types of cancers. Rarely has any study investigated serum miRNAs for multiple different types of cancers for general cancer screening [19–21].

**Fig 1:**
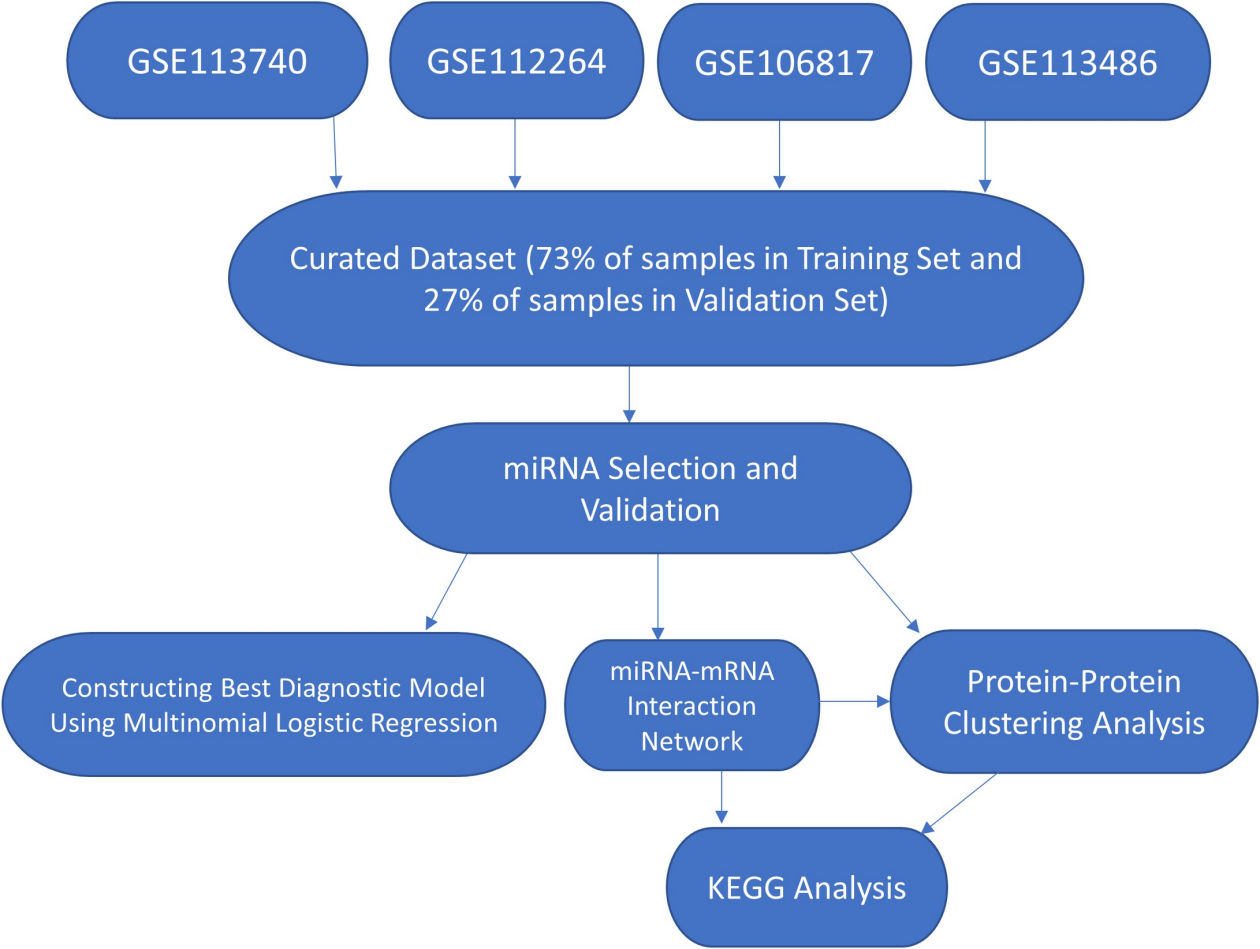
Analysis workflow of the study. The microarray data were manually curated from four studies (GSE113740, GSE112264, GSE106817, GSE113486) and combined for miRNA selection. The selected miRNAs were then used to classify and validate cancer subjects. miRNA-mRNA interaction network, protein-protein clustering analysis, and KEGG analysis were performed.

In this study, we curated large microarray datasets consisting of different types of cancers and non-cancer samples. The cancers include breast, lung, colorectal, prostate, and gastric cancers, which are the top five most prevalent cancers in the world in 2020 [22]. The curated dataset also comprises ovarian and pancreatic cancers, which are well-known to present in late stages [4, 5]. In addition, the dataset includes biliary tract, bladder, liver, and esophageal cancers, gliomas, and sarcomas [14, 16–18]. Candidate miRNAs for general cancer screening for these 13 types of cancers were selected via random forest, a widely used and reliable machine learning algorithm for biomarker discovery [23]. The selected miRNAs were then validated in an independent validation set, and a multinomial logistic regression model was built to distinguish cancer from non-cancer samples. We further investigated the miRNA-mRNA interactions and biological pathways to elucidate the roles these miRNAs may play in cancers. The study workflow is provided in Fig 1.

## Results

### Study design

A curated dataset from four GEO datasets [14, 16–18] yielded 13 different types of cancers and many non-cancer samples. There was a total of 3352 cancer samples and 2809 non-cancer samples. The clinical information of all the samples is detailed in Table 1. The curated dataset was split into a training set with 2253 cancer samples and 2247 non-cancer samples, and a validation set with 1102 cancer samples and 562 non-cancer samples. We used the training set to select promising miRNAs via 100 random forest models and the validation set to verify the selected miRNA as a potential diagnostic marker for cancer detection. The selected miRNAs were then used to perform miRNA-mRNA network analysis, protein-protein interaction clustering analysis, and Kyoto Encyclopedia of Genes and Genomes (KEGG) enrichment analysis [24] (Fig 1).

**Table 1:** Clinical summary for cancer samples.

Cancer type	Mean Age (years)	Standard Deviation (Age in years)	Number of Female: Male	Total Number of Samples	% of Samples in Training	Pathological Stage (if known)
Prostate	67.6	7.5	0:809	809	40%	
Ovarian	56.9	11.5	320:0	320	70%	
Bladder	67.8	10.7	109:283	392	70%	313 with high pathological grade and 77 low pathological grade
Hepatocellular Carcinoma	67.6	9.2	77:268	345	70%	270 child-pugh A, 34 child-pugh B
Breast	55.9	11	155:0	155	80%	
Colorectal	65.7	11	130:75	205	80%	
Sarcoma	53.0	17.6	133:72	205	80%	
Pancreatic	63.5	10.0	130:75	205	80%	
Gastric	66.7	10.0	124:81	205	80%	
Lung	63.8	8.4	126:27	153	80%	
Esophageal	67.3	8.2	119:86	178	80%	
Glioma	52.6	18.6	21:69	90	80%	
Biliary	67.7	9.4	12:78	90	80%	

The pathological stage and grade of many samples were unknown. Many of the patients’ age from which the sample was obtained were unknown. Only 90 samples from sarcomas, colorectal, esophageal, pancreatic, and gastric cancers were used to calculate the mean and standard deviation of age. Only 40 samples from lung and breast cancer were used to calculate mean and standard deviation of age.

### miRNAs selection and validation

**Fig 2:**
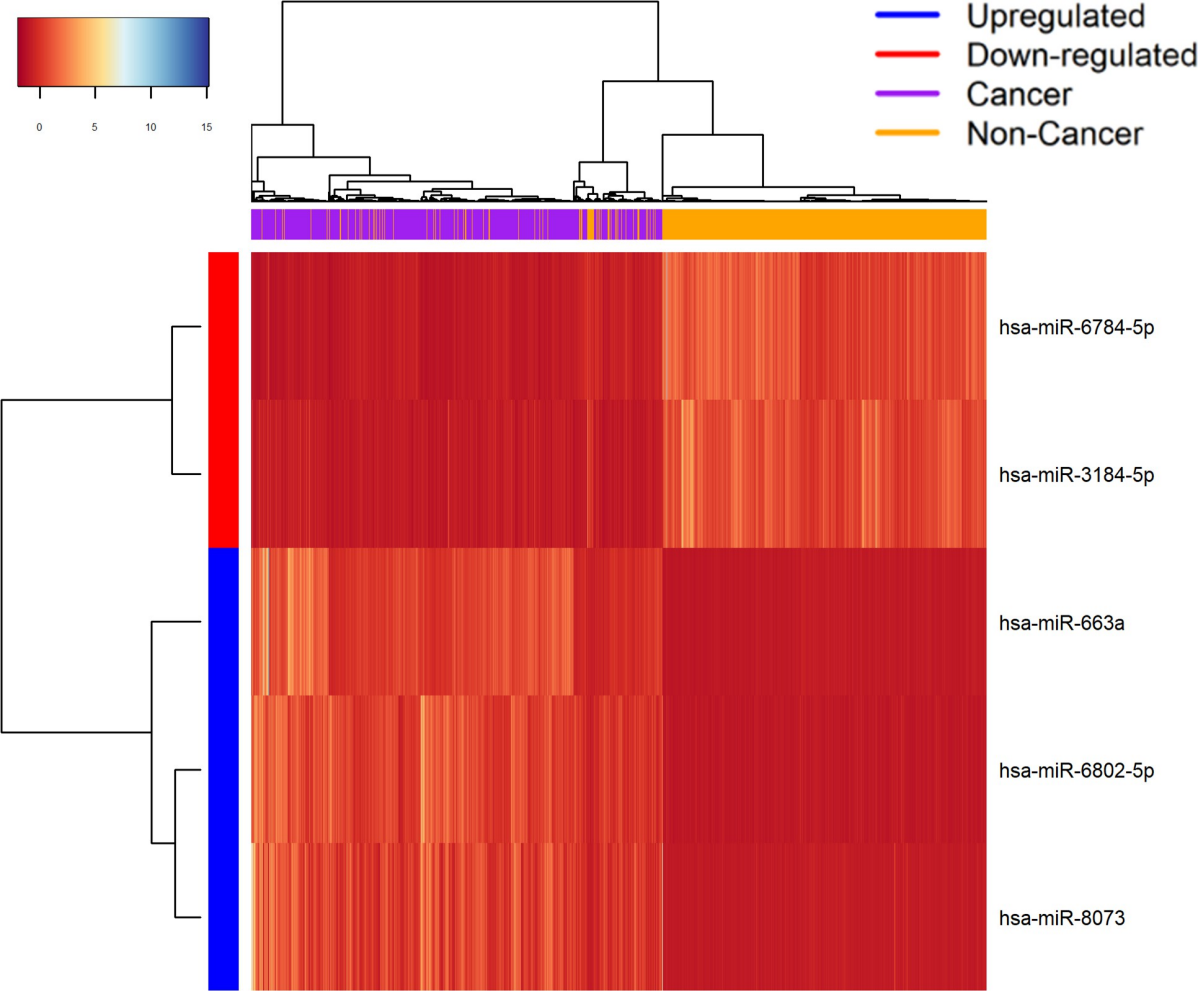
Heatmap of the expression value of the top 5 miRNAs selected from highest frequency miRNAs in 100 random forest models. The X-axis represents the samples, and the Y-axis represents the miRNAs. Each of the boxes represents the normalized expression value of each miRNA in the corresponding sample.

Five miRNAs were considered “balanced”, as they satisfied the criteria of being in the top 10 miRNAs 90% of the time across 100 random forest models [23, 25]. The miRNAs selected were: hsa-miR-3184-5p, hsa-miR-663a, hsa-miR-6784-5p, hsa-miR-6802-5p, and hsa-miR-8073 (Table 2). The random forest models achieved an average of 97% accuracy with 95% bootstrap confidence interval of 0.9544 to 0.9778 [26]. Hierarchical heatmap clustering with complete linkage based on Euclidean distance was performed using these 5 miRNAs across the samples, and the heatmap shows a clear separation between cancer and non-cancer samples [27] (Fig 2).

**Table 2:** Frequency of top miRNAs in 100 random forest models.

miRNA	Frequency
Hsa-miR-3184-5p	100
Hsa-miR-663a	100
Hsa-miR-6784-5p	100
Hsa-miR-6802-5p	96
Hsa-miR-8073	90
Hsa-miR-4783-3p	87
Hsa-miR-1307-3p	86
Hsa-miR-4730	79
Hsa-miR-320a-3p	63
Hsa-miR-5100	45
Hsa-miR-1343-3p	43
Hsa-miR-1469	38
Hsa-miR-1233-5p	30
Hsa-miR-1290	14
Hsa-miR-4675	11
Hsa-miR-1238-5p	8
Hsa-miR-320b	7
Hsa-miR-4532	2
Hsa-miR-4687-5p	1

The table shows the frequency of top miRNAs with respect to highest gini values displayed in the 100 random forest models. The top 5 miRNAs with the highest frequency in this table were chosen to be biomarker candidates for cancer screening.

**Fig 3:**
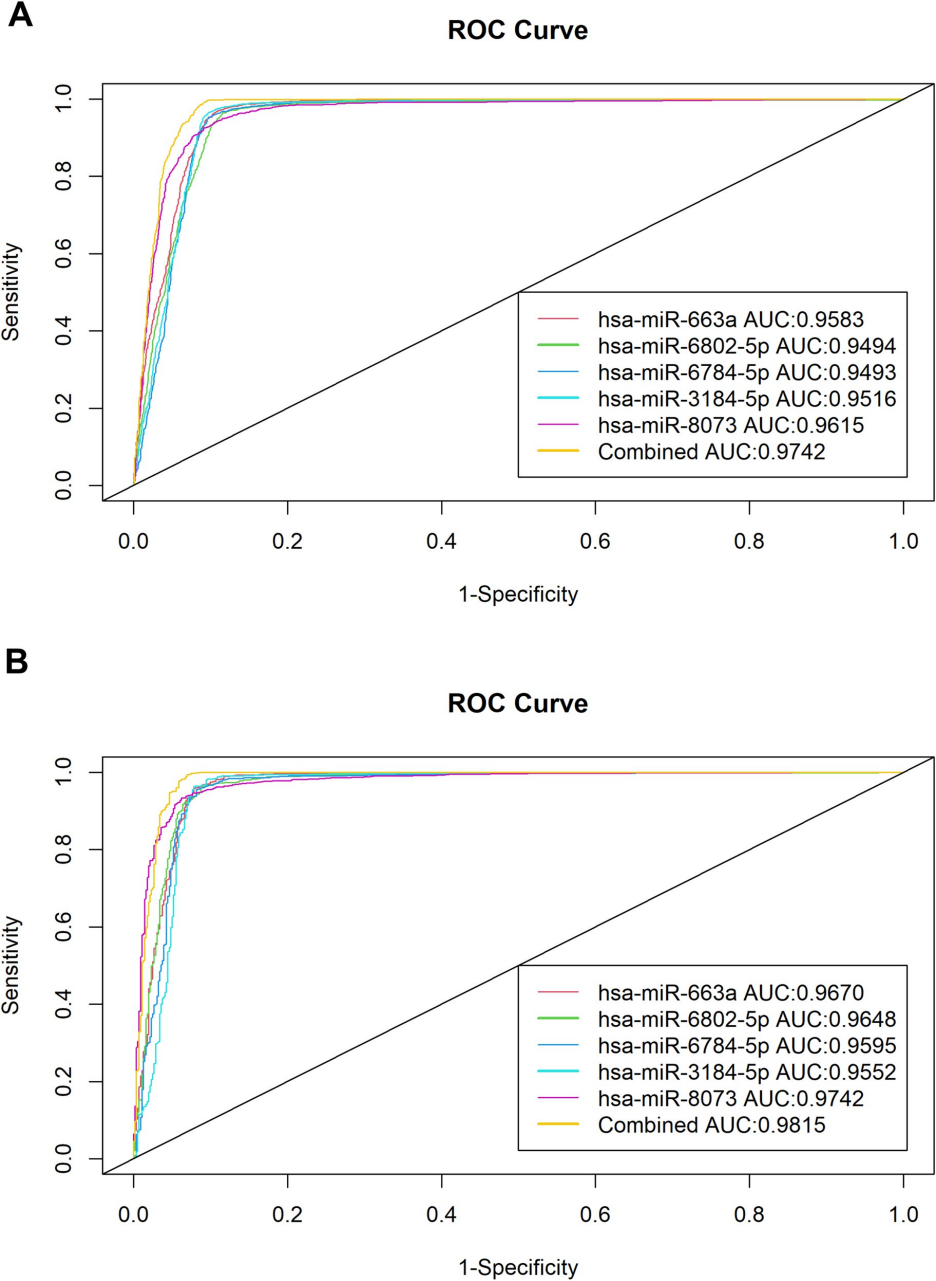
ROC and AUC analysis of the top 5 selected miRNAs and the 4 miRNA combination ROC and AUC values. Panel A is the analysis for the discovery set. Panel B is the analysis for the Validation Set. Both panels achieved the highest ROC and AUC value using 4 miRNAs: has-miR-663a, has-miR-6802, has-miR-3184-5p, and hsa-miR-8073.

The receiver operating characteristic curves and the area under the curve value were used to evaluate the diagnostic potential of each miRNA and their combinations both in the training set and the validation set [28] (Fig 3). Each of the miRNAs showed significant AUC values as displayed in Fig 3 and Table 3. The best combination model based on AUC used only four of the miRNAs: hsa-miR-663a, hsa-miR-6802-5p, hsa-miR-3184-5p, and hsa-miR-8073. The combined model was built using the training set, yielding (0.0005032411) x hsa-miR-663a + (0.0006917428) x hsa-miR-6802-5p + (0.0072807475) x hsa-miR-8073 + (-0.0194274974) x hsa-miR-3184-5p + (-1.1271024323) with AUC value of 0.9742 in the training set. The same model was then used to predict the cancer samples in the validation set, resulting in an accuracy of 0.9652, sensitivity of 0.9773, specificity of 0.9413, and an AUC value of 0.9815. The consistently similar high AUC values across training and validation sets suggest that the models do not overfit.

**Table 3:** Classification statistics of selected miRNAs.

	Accuracy	Sensitivity	Specificity	AUC
All 5 miRNA	0.9687	0.9891	0.9288	0.9780
Hsa-miR-663a, hsa-miR-6802-5p, hsa-miR-3184-5p, Hsa-miR-8073	0.9652	0.9773	0.9413	0.9815
Hsa-miR-663a	0.9453	0.9574	0.9217	0.967
Hsa-miR-6802-5p	0.9435	0.9592	0.9128	0.9648
Hsa-miR-6784-5p	0.9435	0.9555	0.9181	0.9595
Hsa-miR-8073	0.9489	0.9628	0.9217	0.9742
Hsa-miR-3184-5p	0.9351	0.9338	0.9377	0.9552

The table shows classification statistics of each of the 5 selected miRNAs and their best combinations in terms of highest sensitivity and AUC values, which is the combination with all 5 miRNAs and 4 of them (Hsa-miR-663a, Hsa-miR-6802-5p, Hsa-3184-5p, and Hsa-miR-8073), respectively.

### miRNA-mRNA network, functional enrichment, and protein-protein cluster analysis

**Fig 4:**
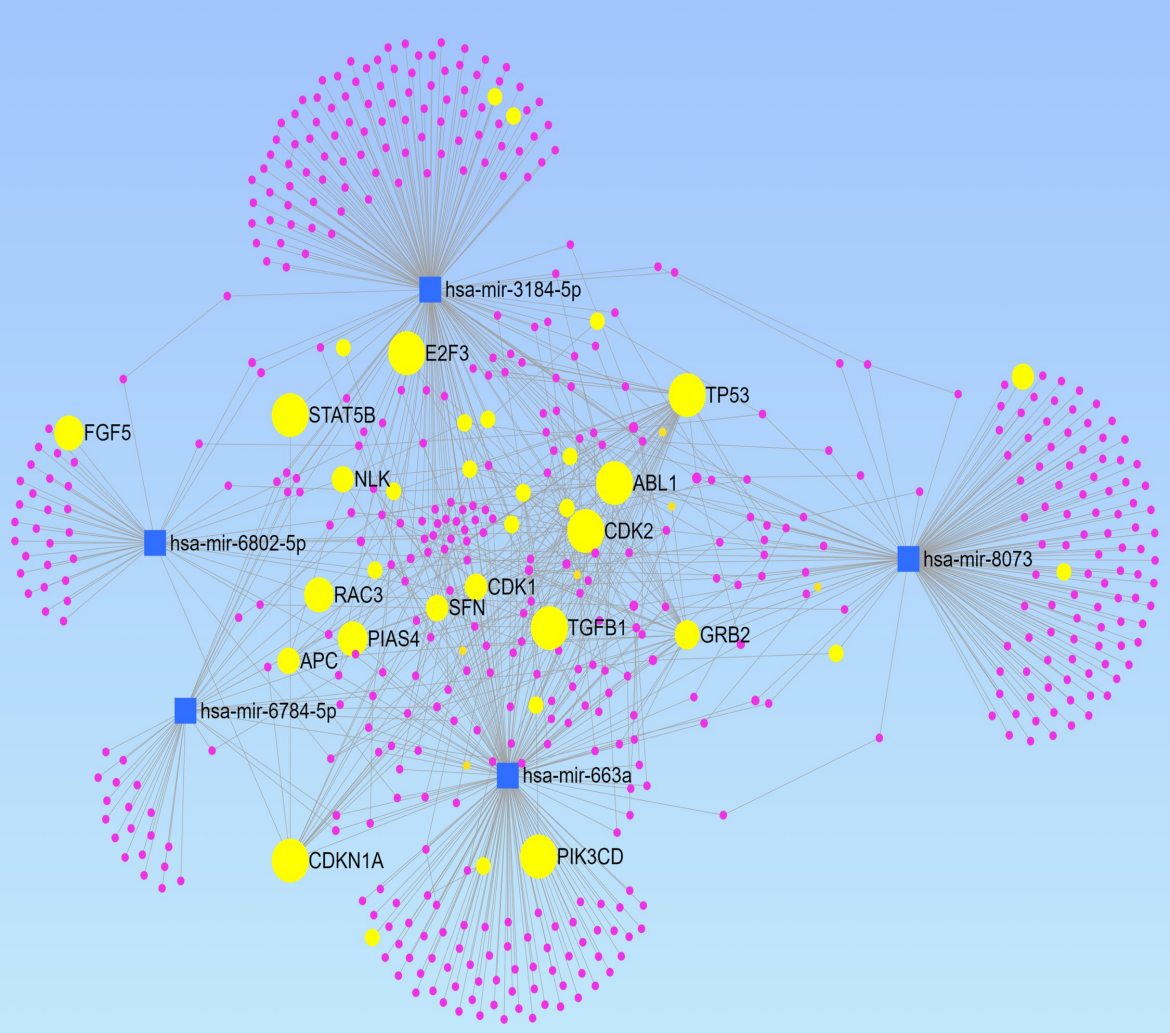
miRNA-mRNA interaction network for the selected 5 miRNAs. The blue squares represent the miRNAs. The purple and yellow circles represent the mRNAs. The yellow circles represent mRNAs directly associated with cancer, with the bigger yellow circles indicating that the mRNA is more associated with the selected 5 miRNAs. The edge between two nodes indicates their interaction.

The network (Fig 4) generated a total of 535 mRNAs, with many of them directly associated with cancer [29]. KEGG analysis of the generated mRNAs was performed. It yielded many significant pathways associated with cancer as well [24] (Table 4), with the most significant one being cell cycle and the second being chronic myeloid leukemia, along with many other cancers, including but not limited to glioma, prostate cancer, bladder cancer, and others. KEGG analysis using the mRNAs and circular RNAs yielded similar results (S1 Table).

**Table 4:** KEGG analysis using mRNAs associated with the 5 selected miRNAs.

Pathways	Hits	P value	Adj. P-value
Cell cycle	12	0.00022	0.01135
Chronic myeloid leukemia	9	0.000227	0.01135
Lysine degradation	7	0.000357	0.0119
Glioma	8	0.000517	0.012925
Neurotrophin signaling pathway	10	0.00282	0.05
p53 signaling pathway	7	0.00331	0.05
Prostate cancer	8	0.0035	0.05
Bladder cancer	4	0.00922	0.11525
HTLV-I infection	12	0.0123	0.1227273
Leukocyte transendothelial migration	8	0.0127	0.1227273
Melanoma	6	0.0135	0.1227273
Fructose and mannose metabolism	4	0.0196	0.1557143
Osteoclast differentiation	8	0.0217	0.1557143
Alcoholism	10	0.0218	0.1557143
Circadian rhythm—mammal	3	0.0247	0.1646667
Endometrial cancer	4	0.0378	0.2347059
ErbB signaling pathway	6	0.0399	0.2347059
MAPK signaling pathway	13	0.0429	0.2383333
Phototransduction	3	0.0463	0.24
Epstein-Barr virus infection	6	0.048	0.24

The table shows the relevant pathways associated with the selected 5 miRNAs based on KEGG analysis using miRnet.

**Fig 5:**
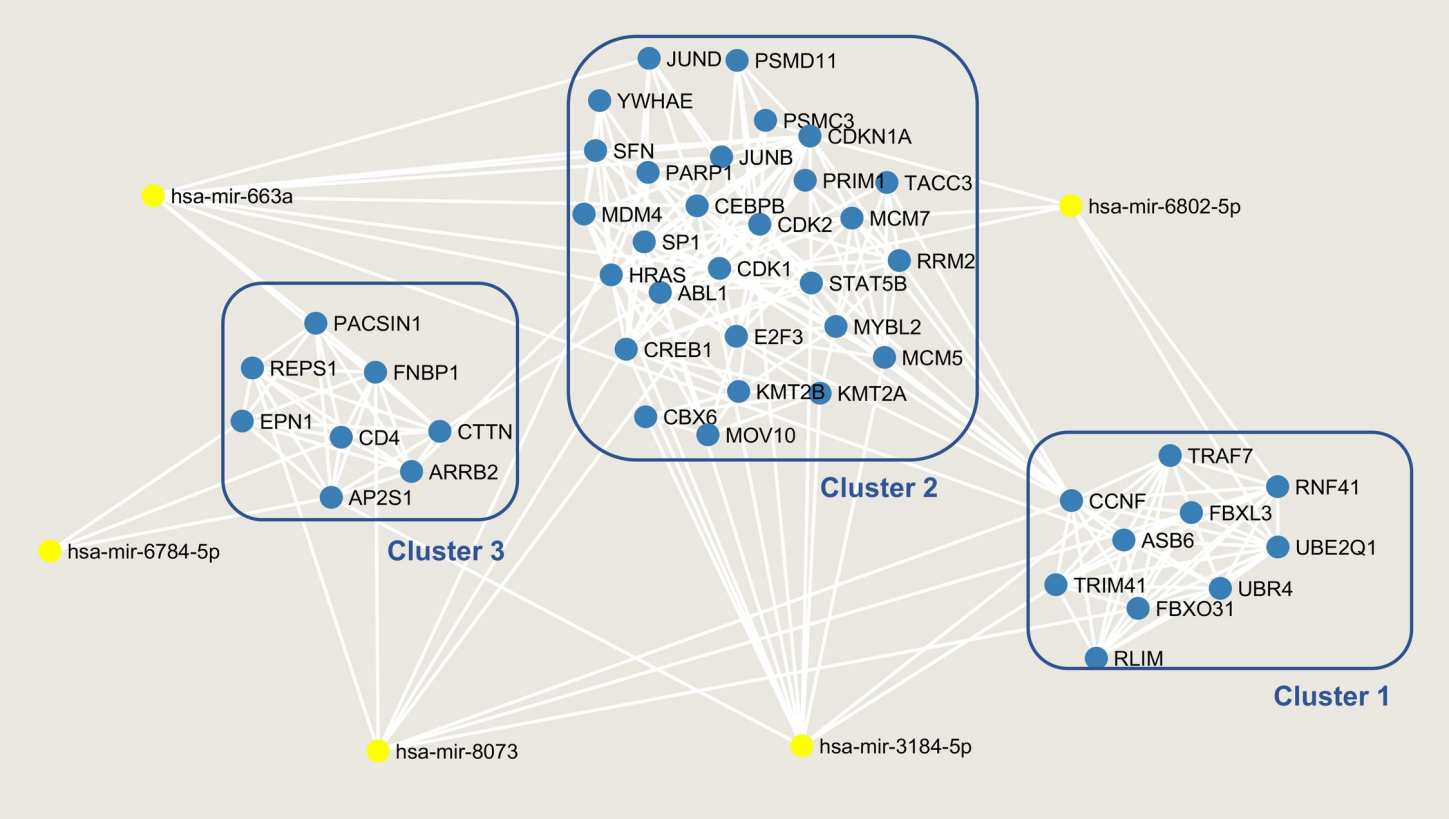
Cluster analysis of the mRNA presented in the miRNA-mRNA interaction network. Top clusters with MCODE value >5 from cytoscape were chosen and the clusters along with their interactions with the 5 selected miRNAs were shown. The miRNAs are highlighted in yellow, and the mRNAs are in blue.

The Protein-Protein-Interaction (PPI) network from the top three resulting clusters yielded 46 nodes, 202 edges, and an average node degree of 8.78 [30]. The PPI enrichment p-value is < 1.0e-16, and KEGG analysis of these proteins yielded many similar pathways with more significant p-values than the previous KEGG analyses (S2 Table). The clusters, along with their interaction with the five chosen miRNAs, are displayed in Fig 5.

## Discussion

Many studies have reported specific upregulation or downregulation of serum miRNAs in certain types of cancers [9, 11–18, 31–35]. Yet, fewer studies have investigated the potential of serum miRNAs as a general cancer screening markers across multiple cancer subtypes [6, 19]. To our knowledge, this is the largest study to assess the potential of miRNAs as markers for general cancer screening, as no other study has combined multiple cancer types to search for general miRNA biomarker for cancer screening.

There are a total of 19 miRNAs identified by random forest models to be important in cancer diagnosis (Table 2); however, only five balanced miRNAs were selected: hsa-miR-663a, hsa-miR-6802-5p, hsa-miR-6784-5p, hsa-miR-3184-5p, and hsa-miR-8073. Consistent with our result, hsa-miR-663a has been reported to regulate cancer signaling and tumor progression; it specifically has been shown to be a sensitive circulating miRNA marker for detection of hepatocellular carcinoma [14, 36]. Similarly, hsa-miR-6784-5p has been reported to be a sensitive serum biomarker for ovarian cancer diagnosis and a key regulator for breast cancer [37]. Hsa-miR-3184-5p is also a key regulator in breast cancer and a reliable biomarker for the early detection of bladder cancer [18, 38]. Interestingly, hsa-miR-8073 is a natural tumor suppressor and identified to be a promising serum biomarker for ovarian and pancreatic cancers [37, 39]. These consistent and overlapping results suggest that these miRNAs may serve as cancer suitable for screening purposes, as other studies also confirmed them to be good biomarkers for specific cancer type. This study reveals that these miRNAs may be nonspecific to a particular cancer, but sensitive across multiple cancers.

Indeed, each of the five miRNAs achieved remarkable results with AUC values well over 0.9 in both training and validation sets, suggesting that these miRNAs may truly be significant and that the models do not overfit. This indicates that the five miRNAs can function as a stand-alone diagnostic marker for at least the 13 types of cancers included in this study, in which some are known for late-stage presentations [4, 5]. Furthermore, the combined model of using four miRNAs: (0.0005032411) x hsa-miR-663a + (0.0006917428) x hsa-miR-6802-5p + (0.0072807475) x hsa-miR-8073 + (-0.0194274974) x hsa-miR-3184-5p + (-1.1271024323) achieved the highest AUC value of 0.9815 in the validation set, which is highly desirable for screening [2]. If sensitivity and accuracy are prioritized, the combination model of using all five miRNAs can be considered to minimize false negatives for screening purposes (Table 3).

miRNA-mRNA network (Fig 4) based on these five miRNAs also further provide evidence that these miRNAs are generally associated with cancers. These miRNAs target many cancer-associated genes including, TP53, ABL1, STAT5B, and E2F3 [40–43] (Fig 4). KEGG analyses also show many enriched cancer-related pathways such as cell cycle, chronic myeloid leukemia, glioma, neurotrophin-signaling, and more [44–46] (Tables 4 and S1 and S2). The top three clusters of mRNAs in the PPI network analysis also pointed toward a very similar result (Fig 5). Many of the same cancer pathways showed up in KEGG with more significant p-values, indicating that these clustered mRNAs are the main actors in enriching these cancer pathways.

There are few limitations to this study. First, the five proposed serum miRNAs have yet to be independently verified. Second, many of the cancer subtypes, cancer stages and grades, and other clinical information are unknown. Though it is possible that many of the samples may represent cancer in the later stages, other studies [14, 18] have shown some of the identified miRNA biomarkers in this study to be valuable in early detection for certain cancers. Nevertheless, due to this lack of information, further studies are warranted to investigate the specificities of each of the proposed miRNAs for early cancer detection and screening. However, the study still demonstrates that these identified miRNAs are useful in cancer detection across multiple cancer types. It also helps elucidate the association of these miRNAs to cancers in general, even if they are not proven effective in early cancer detection. Lastly, despite the effort to balance cancer samples while building the cancer diagnosis model, there are still some imbalances in the number of different cancer types while constructing the model, which may over-represent one cancer over the other.

Overall, the results show high sensitivity and AUC value for the proposed 4-miRNA panel based on highest AUC value. Each individual miRNA achieved significant diagnostic potentials, suggesting that these miRNAs can be used as minimally invasive biomarkers for general cancer screening. Moreover, network and KEGG analyses provided insights into how these miRNAs may play a role in cancer regulation, warranting further investigation. Functional studies of these miRNAs and their associated mRNAs are therefore warranted.

## Materials and methods

### Microarray data processing

GSE113740, GSE112264, GSE106817, GSE113486 datasets were obtained from GEO [14, 16–18]. These datasets were all part of the Japan Initiative to sequence cancer transcriptome via microarray.

As a result, each of these studies was originally part of a larger dataset that was split into smaller datasets for analytical purposes. According to the authors, the presence of miRNA was determined if the signal was greater than the mean + 2X standard deviation of the laboratory’s internal negative control. Then, the background signal was subtracted from each signal that was deemed to be present. As these datasets were generated under the same laboratory, the datasets were normalized with respect to one another using quantile normalization to allow for comparison across all samples. The processed data for analysis is available on the GEO website, and the series matrix files were downloaded from each GEO dataset Since the datasets were originally part of a larger initiative, some of the GEO datasets contained samples that other datasets also had. Therefore, we manually curated the four datasets to ensure there was no duplicated samples while maximizing the number of samples. The curated data contain 13 different types of cancers. The distribution of cancers and the clinical information of the dataset are provided in Table 1. We randomly separated the curated data into training set and validation set before analysis by computer-generated random numbers. To minimize bias over certain cancer types with more samples, we randomly chose 40% of prostate cancers and 70% of ovarian, liver, and bladder cancers in the training set. We randomly chose 80% of the samples in the rest of the cancer types to include in the training set. The remaining samples made up the samples in the validation set. Overall, there were 73% of total samples in the training set and 27% of samples in the validation set. Then, we used the training set to select miRNAs that can successfully screen out cancer samples from normal samples, and the validation set to validate the result. The workflow of this study is provided in Fig 1.

### Balanced miRNA selection and validation

We grouped the different cancer types as cancer samples and compared their expression values to those of the non-cancer samples within the training set. Welch’s t-test was performed for each miRNA, and FDR was calculated. Top 500 miRNAs with the least FDR were chosen to undergo further selection via random forest, a well-known machine learning algorithm often used in studies for cancer classification [23, 47, 48]. Those 500 miRNAs were then put into a further selection process using a total of 100 random forest models [25], with each model randomly selecting 80% of the training set for training, and the remaining 20% for testing. The relevant parameters of the random forest models were optimized using 10-fold cross validation, and the rest of the parameters were mainly set to default. For each model, the mean decrease in gini indices is used to rank how important each miRNA is with regards to classifying cancer and non-cancer samples. Mean decrease in Gini indices is often used a natural feature of random forest classification to rank feature importance; it is calculated as the decrease in impurity of using the feature weighted by the probability of reaching that feature [47, 49]. In each of the 100 random forest models, the miRNAs were deemed “balanced” if they show up as one of the highest 10 miRNAs out of 500 miRNAs in their gini values for over 90 models. Bootstrapping in R using 10,000 replicates was used to calculate the confidence interval of the accuracy achieved by the random forest model [26]. We plotted the hierarchical heatmap of expression values of these miRNAs using complete linkage and Euclidean distance to show separation between cancer and non-cancers [27] (Fig 2).

We then used receiver operating characteristic (ROC) curve analysis and the area under the curve (AUC) to evaluate each miRNA’s potential in distinguishing cancer from normal samples [28]. The curves were generated for both training and validation sets (Fig 3). A higher AUC value indicates a higher distinguishing potential for the miRNA. To improve the discriminating potential even further, multinomial logistic regression model was used to discriminate cancer from the non-cancer samples using combinations of the miRNAs [50]. For transparency and reproducibility purposes, the custom code used in this study is provided under the Availability of Data section.

### MiRNA-mRNA interaction, functional enrichment, and protein-protein interaction analysis

To further study why and how these miRNAs are important in cancer diagnosis, we used miRnet [29] to analyze the relationship between the chosen miRNAs and their associated mRNAs. miRnet [29] is a web-based software that displays all the miRNA-mRNA interactions, providing insight into how these miRNAs might regulate different mRNAs associated with cancer [29]. The analysis was performed with setting organism into homo sapiens and unspecified tissue of origin. Furthermore, two KEGG analyses were performed through miRnet–one using all the associated mRNAs (Table 2) and one with all the circular RNAs in addition to the mRNAs [24, 51] (S1 Table). The default settings for KEGG analyses were used on miRnet, utilizing hypergeometric algorithm and including all associated mRNAs as nodes for Table 2, as well as including all associated mRNAs and circular RNAs as nodes in S1 Table.

The target genes from the miRNA-mRNA interaction network were further clustered to uncover their potential contribution to the development of cancer [29]. The miRNA-mRNA network was uploaded and visualized in the Cytoscape software [52]. Then, the top clusters were chosen using the Molecular Complex Detection (MCODE) technique, with the inclusion criteria of degree cutoff of 2, node score cutoff of 2, k-core of 2, and the maximum depth of 100 [53]. The threshold MCODE score was set to greater or equal to 5 as criteria. The resulting clusters were plotted together as a network. Next, protein-protein interaction analysis was performed by inputting all mRNAs from the top 3 clusters of the miRNA-mRNA network (Fig 5) into the online STRING database v 11 to visualize their interactions [30]. The analysis was done by uploading the list of mRNAs from Fig 5 into the STRING database with the setting of organism as “Homo sapien”. KEGG analysis was also performed using the proteins from the selected clusters using the STRING database website [24, 30] (S2 Table).

## Supporting information

S1 Table KEGG pathways using mRNAs and circular RNAs. Table of enriched KEGG pathways generated from performing KEGG analysis on miRnet using mRNAs and circular RNAs.
(XLSX)

S2 Table KEGG pathways using mRNAs from top 3 clusters. Table of enriched KEGG pathways generated from performing KEGG analysis using mRNAs from top 3 clusters.
(XLSX)
